# Heterogeneous FASnI_3_ Absorber with Enhanced Electric Field for High-Performance Lead-Free Perovskite Solar Cells

**DOI:** 10.1007/s40820-022-00842-4

**Published:** 2022-04-08

**Authors:** Tianhao Wu, Xiao Liu, Xinhui Luo, Hiroshi Segawa, Guoqing Tong, Yiqiang Zhang, Luis K. Ono, Yabing Qi, Liyuan Han

**Affiliations:** 1 State Key Laboratory of Metal Matrix Composites, School of Material Science and Engineering, Shanghai Jiao Tong University, Shanghai, 200240 People’s Republic of China; 2 Special Division of Environmental and Energy Science, Komaba Organization for Educational Excellence (KOMEX), College of Arts and Sciences, University of Tokyo, Tokyo, 153-8902 Japan; 3 Energy Materials and Surface Sciences Unit (EMSSU), Okinawa Institute of Science and Technology Graduate University (OIST), 1919-1 Tancha, Onna-son, Kunigami-gun, Okinawa, 904-0495 Japan; 4 School of Materials Science and Engineering, Henan Institute of Advanced Technology, Zhengzhou University, Zhengzhou, 450001 People’s Republic of China

**Keywords:** Gradient FASnI_3_ absorber, Built-in electric field, Bulk charge recombination, Lead-free perovskite solar cell

## Abstract

Lead-free tin perovskite solar cells (PSCs) have undergone rapid development in recent years and are regarded as a promising eco-friendly photovoltaic technology. However, a strategy to suppress charge recombination via a built-in electric field inside a tin perovskite crystal is still lacking. In the present study, a formamidinium tin iodide (FASnI_3_) perovskite absorber with a vertical Sn^2+^ gradient was fabricated using a Lewis base-assisted recrystallization method to enhance the built-in electric field and minimize the bulk recombination loss inside the tin perovskites. Depth-dependent X-ray photoelectron spectroscopy revealed that the Fermi level upshifts with an increase in Sn^2+^ content from the bottom to the top in this heterogeneous FASnI_3_ film, which generates an additional electric field to prevent the trapping of photo-induced electrons and holes. Consequently, the Sn^2+^-gradient FASnI_3_ absorber exhibits a promising efficiency of 13.82% for inverted tin PSCs with an open-circuit voltage increase of 130 mV, and the optimized cell maintains over 13% efficiency after continuous operation under 1-sun illumination for 1,000 h.

**Supplementary Information:**

The online version contains supplementary material available at 10.1007/s40820-022-00842-4.

## Highlights

- A novel strategy to further improve the efficiency of lead-free tin perovskite solar cells by carefully controlling the built-in electric field in the absorber is described.
- A promising efficiency of 13.82% was obtained based on the formamidinium tin iodide (FASnI_3_) perovskite solar cells with a vertical Sn^2+^ gradient and an enhanced electric field.
- The solar cell with a heterogeneous FASnI_3_ absorber is ultrastable, maintaining over 13% efficiency after operation under 1-sun illumination for 1,000 h in air.

**Supplementary Information:**

The online version contains supplementary material available at 10.1007/s40820-022-00842-4.

## Graphical abstract

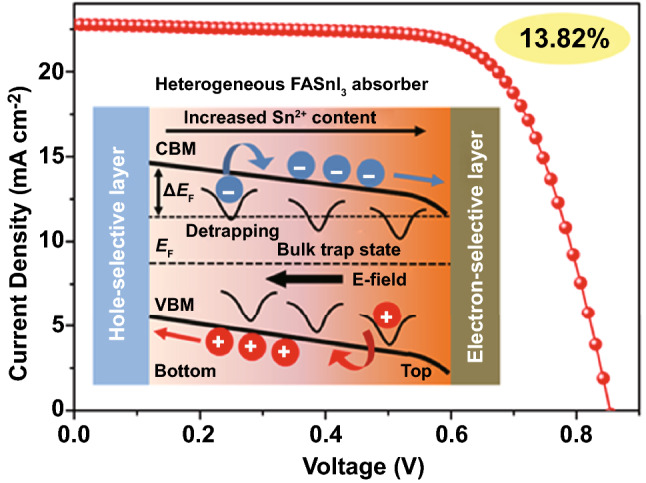

## Introduction

Lead-free tin halide perovskites with eco-friendly properties, high carrier mobility, and a suitable bandgap close to the Shockley–Queisser limit are regarded as promising candidates for developing next-generation perovskite solar cells (PSCs) [1–6]. In recent years, the power conversion efficiency (PCE) of tin PSCs based on the formamidinium tin iodide (FASnI_3_) perovskite absorber has increased to over 10% [7–11]. One important reason for this rapid PCE growth is an increase in carrier diffusion length in the tin perovskite layer. This has been enabled by introducing large organic cations such as n-butylammonium (BA), phenylethylammonium (PEA), guanidinium (GA), and pentafluorophen-oxyethylammonium (FOE) to form a low-dimensional tin perovskite phase and produce a highly oriented polycrystalline film [12–17] or by introducing reducing agents to eliminate Sn^4+^ impurities, such as tin halides (SnX_2_), metallic Sn, hydroxybenzene sulfonic acid, and formic acid [18–22]. Furthermore, the architecture of tin PSCs has evolved from a regular (n-i-p) to an inverted (p-i-n) structure to avoid oxidization of Sn^2+^ by the chemical dopants of hole transport materials used in the n-i-p structure, resulting in better device stability [23–25].

After the PCE reached 10%, many studies focused on the surface passivation of the tin perovskite layer to minimize the deficit between the bandgap and open-circuit voltage (*V*_OC_) in tin PSCs [26–29]. For example, an edamine with a lone electron pair was applied to passivate the FASnI_3_ perovskite surface via a coordination reaction with the unsaturated Sn^2+^, resulting in 50% enhancement of the carrier lifetime and increased *V*_OC_ of tin PSCs by 100 mV to 0.6 V [30]. Similarly, gallic acid, hydroxybenzene sulfonic acid, and thiosemicarbazide molecules with coordination functional groups have also been applied as surface passivation agents for tin PSCs to increase the photovoltage [21, 26, 31]. Additionally, a p-type semiconducting polymer with amine groups was used to passivate the bottom interface of the FASnI_3_ absorber and accelerate hole extraction in the inverted tin PSCs, resulting in a large *V*_OC_ enhancement [32]. Moreover, recently, a wide-bandgap two-dimensional anilinium-based tin perovskite capping layer was constructed atop the FASnI_3_ absorber via sequential solution deposition method, yielding an increase of the carrier lifetime by about 20 times and a high *V*_OC_ approaching 0.7 V [15]. In addition to occurring at the surface, charge recombination occurring inside the tin perovskite crystal seems to be a more critical limitation for *V*_OC_ improvement because the bulk defects of tin perovskite are two orders of magnitude (10^15^‒10^16^ cm^−3^) larger than those of lead perovskite (10^13^‒10^14^ cm^−3^) [27]. However, a strategy to suppress charge recombination in the bulk of the tin perovskite is still lacking. Therefore, advanced bulk passivation technology for tin PSCs should be employed to further improve its efficiency.

Enhancing the built-in electric field in the light-absorbing layer is regarded as an efficient way to reduce charge recombination in the bulk for many thin-film photovoltaic technologies such as organic solar cells, quantum dot solar cells, and Cu(In,Ga)Se_2_ (CIGS) solar cells [33–37]. For tin halide perovskite absorbers, a Sn^2+^-poor condition causes a downshift of the Fermi level (*E*_F_) and enables a unipolar p-type characteristic with a very high hole concentration, while a Sn^2+^-rich condition effectively increases the electron density and tunes the characteristics of tin perovskite from p-type to an intrinsic or relatively n-type semiconductor with a higher *E*_F_ position [38–43]. This unique self-doping property allows the production of a localized *E*_F_ difference inside the tin perovskite absorber by realizing a heterogeneous distribution of Sn^2+^ to enhance the electric field and further reduce the bulk recombination loss in tin PSCs.

To date, creating a vertical halide gradient (I and Br) or organic cation gradient (phenethylammonium and guanidinium) has been reported for tuning the vertical band structure and *E*_F_ alignment in nanoscale-thickness perovskite absorbers, leading to improved efficiency of PSCs [44–46].

Herein, we describe a FASnI_3_ absorber fabricated with a Sn^2+^ gradient in the vertical direction to minimize carrier recombination loss in the bulk and further improve the device efficiency by enhancing the built-in electric field. This vertical Sn^2+^ gradient was realized by recrystallization of the as-prepared FASnI_3_ perovskite assisted by a polymethyl methacrylate (PMMA) layer inserted on the upper surface. During the recrystallization process, the carboxylate groups on PMMA could form strong coordination bonds with the Sn^2+^ cations and enrich the Sn^2+^ on the FASnI_3_ surface. Time-of-flight secondary ion mass spectrometry (Tof–SIMS) and depth-dependent X-ray photoelectron spectroscopy (XPS) revealed that *E*_F_ increases with increasing Sn^2+^ content from the bottom to the top in the heterogeneous FASnI_3_ film, leading to an additional electric field to assist charge detrapping from bulk defects and accelerate the oriented separation of photo-induced electrons and holes. Consequently, this Sn^2+^-gradient FASnI_3_ absorber boosts the PCE of inverted tin PSCs from 10.89 to 13.82% with a *V*_OC_ increase of 130 mV, and the optimized device still shows a PCE of over 13% after operation under 1-sun illumination for 1,000 h. This study provides a promising route to further improve the PCE of tin PSCs by carefully controlling the built-in electric field in the tin perovskite absorbers.

## Experimental Section

### Tin PSC Fabrication

The indium tin oxide (ITO) glass substrates were first patterned and cleaned by sequential ultrasonic cleaning with acetone and isopropanol for 10 min and then treated with a UV-ozone cleaner for 30 min before the deposition of polyethylene glycol-poly(3,4-ethylenedioxythiophene):polystyrene sulfonate (PEG-PEDOT:PSS) as the hole transport layer (HTL), as demonstrated in our previous report [47]. To prepare the perovskite precursor solution, formamidinium iodide (FAI), ethylenediammonium diiodide (EDAI_2_), SnI_2,_ and SnF_2_ were added to the dimethyl sulfoxide (DMSO) solvent at a molar ratio of 0.99: 0.005: 1: 0.06, with a total concentration of 0.9 M. Then, the perovskite precursor solution was spin-coated onto the ITO glass at 1,000 rpm for 10 s and then 5,000 rpm for 50 s, after which chlorobenzene anti-solvent was dripped onto the tin perovskite film for 30 s. The resulting films were then annealed at 100 °C for 12 min. Subsequently, 0.005 mg mL^−1^ PMMA in chlorobenzene was spin-coated onto the FASnI_3_ layer at 3,000 rpm. For the recrystallization process, the as-prepared FASnI_3_ film was placed on a hotplate (60 °C) covered with a petri dish, and 50 μL DMSO was dropped near the film at a distance of 1 cm. Finally, C_60_ (45 nm), bathocuproine (BCP, 5 nm), and an Ag electrode (80 nm) were sequentially evaporated onto the tin perovskite layer under a high vacuum of 2 × 10^–5^ Pa.

### Film Characterization

Scanning electron microscopy (SEM) images and energy-dispersive X-ray spectroscopy (EDS) were obtained using a Hitachi SU8000 field-emission scanning electron microscope (Japan). To prevent interference from extra elemental Sn signals from the ITO substrate, the FASnI_3_ films were fabricated on a silicon substrate for Tof–SIMS and EDS measurements. Tof–SIMS measurements were performed using a focused ion-beam Tof–SIMS spectrometer (GAIA3 GMU Model, Czech Republic). A 30 keV Bi^+^ ion beam was used as the primary ion beam to peel the samples with an analysis area of 5 × 5 µm^2^. X-ray diffraction (XRD) patterns were measured on a Rigaku powder X-ray diffractometer (Japan) using a Cu K*α* radiation source at a scan rate of 5° min^−1^. UV–Vis absorption spectra of the tin perovskite films were recorded using a Shimadzu spectrometer (Japan). Time-resolved photoluminescence (TRPL) analysis was performed using a Hamamatsu C12132 fluorescence lifetime spectrometer (Japan) at an excitation wavelength of 450 nm. Photoelectron spectroscopy in air (PESA) was performed using a Riken Keiki AC-3 instrument (Japan). XPS analysis was performed on a Kratos AXIS Ultra DLD spectrometer (UK) with an Al K*α* X-ray source, and an Ar^+^ source was used to etch the tin perovskite film for detecting the depth profiles of *E*_F_. Grazing incident X-ray diffraction (GIXRD) patterns were measured with a Bruker powder X-ray diffractometer (Germany) using Cu K*α* radiation. Scanning Kelvin probe microscopy (SKPM) measurements were performed by an atomic force microscope (AFM, Asylum Research MFP-3D, Oxford Instruments) using Ti/Ir coated Si cantilevers with a nominal spring constant of 2 N m^−1^ (Model: ASYELEC-01). For the SKPM measurements, a Nap mode was used with a DC bias voltage applied to the AFM tip to determine the contact potential difference between the AFM tip and the sample surface.

### Device Characterization

Dark current curves were measured using a DC source monitor (ADC Corporation, 6244, Japan). The *J–V* curves of the tin PSCs were measured using a solar simulator with a standard air mass of 1.5 sunlight (100 mW cm^−2^, WXS-155S-10, Wacom Denso, Japan). The active area was defined with a 0.09 cm^2^ mask. Monochromatic incident photon-to-current efficiency (IPCE) spectra were measured with monochromatic incident light (1 × 10^16^ photons cm^−2^) in the director current mode (CEP-2000BX, Bunko-Keiki). The light intensity of the solar simulator was calibrated using a standard silicon solar cell. The Mott–Schottky curves of the tin PSCs were measured on a multifunctional electrochemical workstation (Zahner, Germany), with a constant scanning voltage step of 20 mV. All tin PSCs that underwent the stability test were encapsulated according to the process described in our previous study [48]. First, the perovskite from the working area was wiped out using a mixed solvent of dimethylformamide and methanol; then, the cells in N_2_ were encapsulated by cavity glass with UV glue on the edges of the working area. Finally, the UV glue was fixed under UV light for 20 s.

## Results and Discussion

### Construction of the FASnI_3_ Absorber with Vertical Sn^2+^ Gradient

**Fig. 1:**
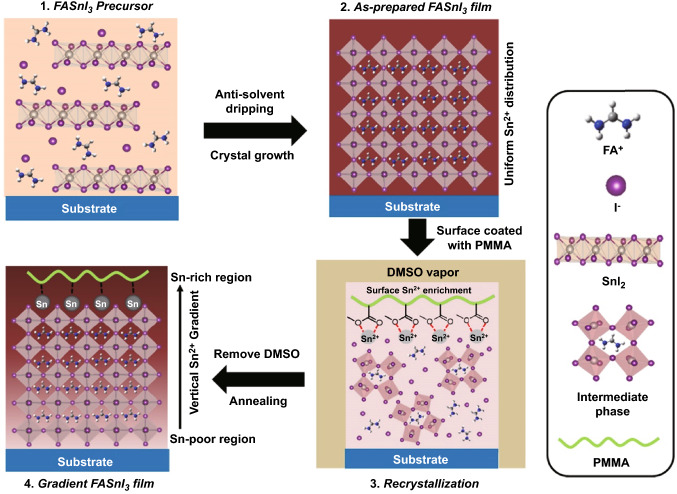
Schematic of the fabrication of gradient FASnI_3_ film with an increased Sn^2+^ content from bottom to top by recrystallization of the as-prepared FASnI_3_ perovskite film assisted with surface-coated PMMA with a high concentration of carboxylate groups

The fabrication process of the FASnI_3_ absorber with a vertical Sn^2+^ gradient is illustrated in Fig. 1. In the first step, the FASnI_3_ precursor solution was dropped onto the substrate, and a certain amount of EDAI_2_ was employed as an additive to improve the crystallinity and reduce the pinholes of the FASnI_3_ perovskite [49]. After the induction of crystal growth thermal annealing, a FASnI_3_ film with a uniform Sn^2+^ distribution is supposed to be formed. Subsequently, we spin-coated a Lewis base-rich polymer, PMMA, on the surface and then treated the as-prepared PMMA-coated FASnI_3_ film with DMSO vapor to form an intermediate wet film, leading to recrystallization of the FASnI_3_ perovskite. In the FASnI_3_-DMSO intermediate film, Sn^2+^ tends to be enriched on the top surface because of the strong coordination interactions formed between the carboxylate groups (O=C–O) and Sn^2+^ cations [50], resulting in a gradient Sn^2+^ distribution in the vertical direction after the second annealing process. In the following discussion, we denote the FASnI_3_ film without any modification as “FASnI_3_” control and the FASnI_3_ film with both PMMA coating and DMSO-vapor treatment as “FASnI_3_ gradient.” For comparison, we also prepared an FASnI_3_ film with PMMA coating but without DMSO-vapor treatment (denoted as PMMA coating) and a film with DMSO-vapor treatment but without PMMA coating (denoted as DMSO-vapor treatment).

**Fig. 2:**
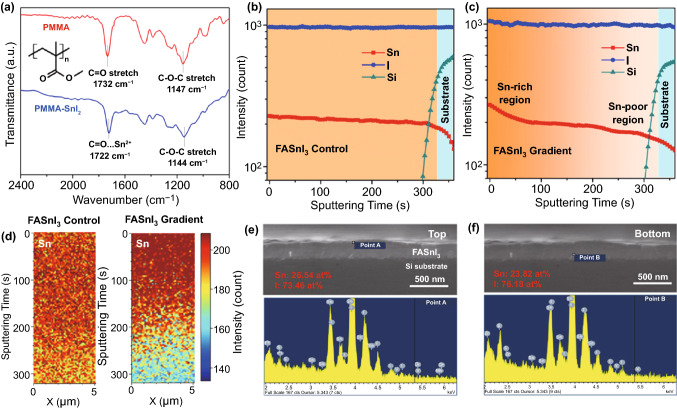
**a** FTIR spectroscopy of the pure PMMA and PMMA-SnI_2_ mixture. Tof–SIMS depth profiles of the Sn, I, and Si elements in **b** FASnI_3_ control and **c** FASnI_3_ gradient films coated on the silicon substrate. **d** Tof–SIMS two-dimensional mapping profiles of Sn along the *x–z* plane for the FASnI_3_ control and FASnI_3_ gradient films. Cross-sectional SEM images and EDS point profiles derived from the **e** top and **f** bottom of gradient film

The coordination interaction between Sn^2+^ and the carboxylate group was confirmed using Fourier transform infrared (FTIR) spectroscopy. As shown in Fig. 2a, both the vibration signals of the C=O and C–O–C groups shifted to a smaller wavenumber after being mixed with the SnI_2_ precursor, attributed to the deformation of the electron cloud and reduced bond stiffness induced by the formation of bidentate coordination. After thermal annealing, the intermediate phase with PMMA is expected to form a solid-state FASnI_3_ film with a gradient increase in elemental Sn from the bottom to the surface.

The reason for choosing PMMA as the Sn^2+^-enrichment additive is that each PMMA monomer contains a carboxylate group that could form bidentate coordination with Sn^2+^, and such high-concentration Lewis base groups could extract a certain amount of Sn^2+^ to the surface in the FASnI_3_ wet film during recrystallization. Furthermore, unlike other small-molecule additives, the large-size PMMA cannot permeate into the bottom of the FASnI_3_ film, which ensures that the Sn-enrichment process only occurs at the top surface of the film.

First, we investigated the effect of the DMSO-induced recrystallization process on the surface morphology of the FASnI_3_ perovskite films. Figure S1 shows top-view SEM images of the FASnI_3_ films after exposure to DMSO vapor for different times. It can be observed that the FASnI_3_ film maintained a compact polycrystalline morphology when the exposure time was less than 5 s. In contrast, a longer exposure time led to many pinholes being formed at the grain boundary, and an island-like structure was formed after 15 s exposure to DMSO vapor, indicating serious corrosion of the FASnI_3_ crystal by DMSO vapor. In addition, the XRD patterns of the intermediate film (gray color) after 5 s DMSO exposure and the film after the second annealing (black color) are depicted in Fig. S2a; the intermediate film exhibited an unordered amorphous structure without obvious diffraction peaks, and the peak at 14.0° (perovskite phase) appeared after the re-annealing process, which confirmed that the recrystallization process had occurred. Then, we measured the XRD patterns of the FASnI_3_ films undergoing recrystallization with different DMSO exposure times to understand the effect of DMSO vapor on crystallinity, as shown in Fig. S2b. It was found that the FASnI_3_ film treated with DMSO vapor for up to 5 s maintained the same intensity of the (100) and (200) diffraction peaks at 14.0° and 28.2° compared to the fresh sample, but a longer DMSO treatment time significantly reduced the film crystallinity. Consequently, we chose an exposure time of 5 s for the recrystallization process to fabricate the gradient FASnI_3_ perovskite film in subsequent studies.

### Vertical Composition Analysis of the FASnI_3_ Gradient Film

To investigate the vertical elemental distribution, we measured the Tof–SIMS depth profiles of the control and gradient films deposited on a Si substrate to avoid the effect of the outside Sn signal from the ITO glass. As shown in Fig. 2b, the intensities of the Sn and I signals remained almost the same in the vertical direction in the control sample, indicating that the Sn^2+^ distribution was homogeneous. In contrast, the intensity of the Sn signal in the gradient film increased gradually during the first 50 s of sputtering time, whereas the I signal did not show any obvious change (Fig. 2c), indicating that a Sn-rich region formed at the upper part and a Sn-poor region at the bottom (from 250 to 330 s). Moreover, Tof–SIMS two-dimensional mapping profiles of elemental Sn along the *x–z* plane were applied to visualize the top Sn-rich region (indicated by dark red) and the bottom Sn-poor region (indicated by light blue) in the FASnI_3_ gradient film (Fig. 2d). In contrast, the control FASnI_3_ film exhibited a uniform color-mapping profile. We then calculated the top and bottom Sn/I ratios in the FASnI_3_ gradient film using energy-dispersive spectrometry (EDS). As shown in Fig. 2e, the top Sn/I ratio was calculated to be 1.08:3 according to the EDS point profile, which is larger than 1:3 in the ideal FASnI_3_ perovskite crystal. In contrast, the bottom Sn/I ratio was calculated to be 0.94:3 (Fig. 2f), indicating that a Sn-poor condition was formed in the bottom region. For comparison, we also estimated the Sn/I ratio of the control film using EDS analysis (Fig. S3a-b). The top and bottom Sn/I ratios were calculated to be 1.04:3 and 1.03:3, respectively, indicating homogeneous elemental distribution in the pristine FASnI_3_ film. We consider that the deviation of the Sn/I ratio from the ideal value (1:3) in the FASnI_3_ control film is ascribed to the presence of extra SnF_2_, which is a commonly used antioxidant additive in high-performance tin PSCs [51].

Subsequently, GIXRD analysis was conducted to study the effect of the vertical compositional gradient on the lattice parameters of the FASnI_3_ films. As shown in Fig. S4, no obvious change in the position of the (100) diffraction peak was found in the FASnI_3_ gradient film when the X-ray incident angle increased from 0.2° to 1.0°, which means that the self-doping effect of excess Sn^2+^ or Sn vacancies did not cause a significant lattice distortion of the corner-sharing FASnI_3_ perovskite crystal, reducing the phase stability.

### Optical and Electronic Property of the FASnI_3_ Gradient Film

We then studied the effect of this vertical Sn^2+^ gradient on the optical and electronic properties of the FASnI_3_ perovskite films. As can be seen in Fig. S5a, the FASnI_3_ gradient sample exhibited a similar absorption in the visible light region to the control sample, indicating that the self-doping effect had a negligible influence on the light-harvesting ability of FASnI_3_ absorbers. Based on the Tauc plots in Fig. S5b, the bandgaps of the FASnI_3_ control and gradient films were calculated to be 1.42 and 1.41 eV, respectively, which were consistent with those reported in a previous study [50]. We also investigated the effect of Sn-rich and Sn-poor conditions on the bandgap of FASnI_3_ films by fabricating a FASnI_3_ film with excess SnI_2_ (7.7 mol%) and FAI (6.3 mol%) to reach the same Sn/I stoichiometric ratio of the top and bottom regions of FASnI_3_ gradient film. Both the Sn-rich and Sn-poor FASnI_3_ films showed a bandgap at approximately ~ 1.41 to 1.42 eV, indicating negligible bandgap fluctuation (less than 0.01 eV) in the vertical direction of the FASnI_3_ gradient film. Subsequently, we measured the valence band maximum (VBM) using PESA to understand the band structure of the FASnI_3_ control and gradient films. As shown in Fig. S5c, the VBM of control and gradient samples were calculated to be − 4.91 and − 4.93 eV, respectively. In addition, the PESA plots in Fig. S5d confirmed that the Sn-rich and Sn-poor conditions did not influence the VBM position of the FASnI_3_ gradient perovskite. After deducing the bandgap value, we calculated the conduction band minimum (CBM) of the control and gradient films to be − 3.49 and − 3.51 eV, respectively.

**Fig. 3:**
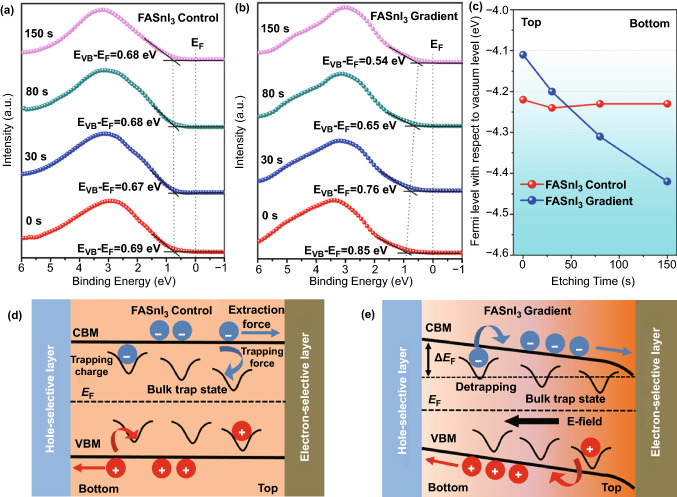
The valence-band XPS spectra of the **a** FASnI_3_ control and **b** FASnI_3_ gradient films with different Ar^+^ etching time. **c** Depth profiles of the *E*_F_ in FASnI_3_ control and FASnI_3_ gradient films derived from the valence-band XPS analysis. Predicted energy-level diagram and charge trapping model in the **d** FASnI_3_ control and **e** FASnI_3_ gradient films in contact with the electron-selective and hole-selective layers

Furthermore, we used an Ar^+^ source to etch the FASnI_3_ films and measured the *E*_F_ at different etching times via valence-band XPS analysis to study the depth profile of the Fermi level. As shown in Fig. 3a, the distance between VBM and *E*_F_ was calculated to be approximately ~ 0.67 to 0.69 eV for the entire FASnI_3_ control film. By contrast, this value decreased from 0.85 (top) to 0.54 eV (bottom) in FASnI_3_ gradient film, indicating an *E*_F_ difference of about 0.3 eV in the monolithic perovskite film (Fig. 3b). To illustrate this vertical *E*_F_ gradient, we derived the depth profiles of the *E*_F_ shown in Fig. 3c using valence-band XPS analysis. It can be seen that the *E*_F_ with respect to the vacuum level of control film locate at approximately − 4.20 to − 4.22 eV, associated with an intrinsic-semiconductor characteristic without extra doping. In the case of FASnI_3_ gradient film, a gradual decrease of *E*_F_ from − 4.11 to − 4.42 eV was found, indicating n-type doping at the top region and p-type doping at the bottom region regarding the constant VBM and CBM values in the vertical direction of the gradient film. In addition, it is worth noting that the *E*_F_ change at the top was larger than that at the bottom in the FASnI_3_ gradient film, which was consistent with the trend of the vertical Sn^2+^ distribution derived from the Tof–SIMS measurement.

Based on this *E*_F_ depth profile, we drew a possible band structure and charge trapping model for the FASnI_3_ control (Fig. 3d) and gradient (Fig. 3e) absorbers. For the control film, no obvious band bending was formed owing to the homogeneous *E*_F_ distribution, and the charge extraction force mainly came from the energy offset at the electron-selective or hole-selective interfaces. In this regard, carriers inside the perovskite crystal can be easily trapped by the bulk defects. In contrast, the *E*_F_ difference (△*E*_F_) in the FASnI_3_ gradient film caused downward band bending at the top electron-selective interface and upward band bending at the bottom hole-selective interface, generating an extra built-in electric field in the FASnI_3_ absorber to retard charge trapping by the bulk defects and promote oriented charge transport and extraction, resulting in a lower recombination loss inside the FASnI_3_ absorber [52]. However, the surface high-resolution C 1* s* XPS spectra (Fig. S6) showed additional peaks at 287.1 eV (C–O–C group) and 288.0 eV (O=C–O group) in the FASnI_3_ gradient sample [53], indicating the presence of PMMA on the surface after recrystallization, which could further induce surface passivation to reduce non-radiative recombination loss [54, 55].

### Carrier Separation Dynamics in the FASnI_3_ Gradient Film

**Fig. 4:**
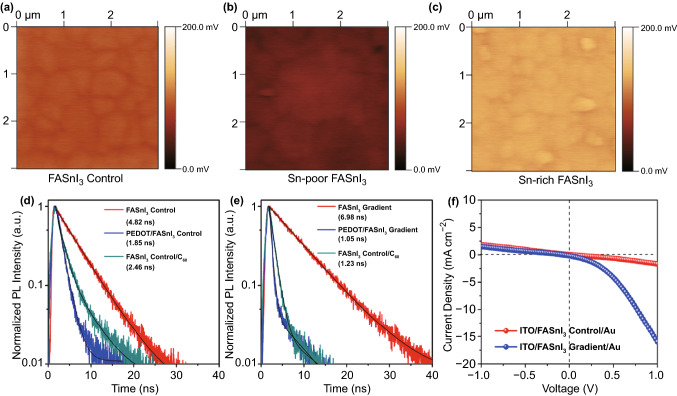
Surface potential mapping images of the **a** control, **b** Sn-poor, and **c** Sn-rich FASnI_3_ perovskite films deposited on the PEDOT:PSS/ITO substrate. TRPL spectra of the **d** control and **e** gradient FASnI_3_ films coated on the quartz substrate before and after contact with C_60_ or PEDOT:PSS layers. **f** Output dark current plots as a function of the applied voltage for the ITO/FASnI_3_ control/Au and the ITO/FASnI_3_ gradient/Au devices

SKPM was used to characterize the surface potential difference of the FASnI_3_ perovskite under Sn-rich and Sn-poor conditions to further illustrate the Fermi level difference induced by the tin source gradient. As shown in Figs. 4a‒c and S7, the control film exhibited an almost uniform surface potential distribution with an AFM tip DC voltage of approximately 100 mV, while a lower AFM tip DC voltage of approximately 50 mV was found for the Sn-poor FASnI_3_ perovskite (6.3 mol% FAI excess), which can be attributed to hole accumulation that causes a downward shift of the Fermi level towards the valence band. In contrast, for the Sn-rich film (7.7 mol% SnI_2_ excess) a higher AFM tip DC voltage of approximately 125 mV was observed because of the n-type doping effect, which was consistent with the trend found in the XPS results. Subsequently, we compared the charge extraction capability of the FASnI_3_ control and FASnI_3_ gradient films by calculating the decay lifetime of the TRPL spectra (Fig. 4d–e). The average decay lifetime (*τ*_ave_) was calculated using the double-exponential decay equation, and the fitting parameters are summarized in Table S1. We found that the *τ*_ave_ of the control film decreased from 4.82 to 1.85 ns and 2.46 ns after the incorporation of HTL PEDOT:PSS and electron transport layer (ETL) fullerene C_60_, respectively. By contrast, the *τ*_ave_ values of FASnI_3_ gradient, PEDOT/FASnI_3_ gradient, and FASnI_3_ gradient/C_60_ films were calculated to be 6.98, 1.05, and 1.23 ns, respectively. To understand the increased *τ*_ave_ in the gradient film, we measured the TRPL plots of the FASnI_3_ film with a coating of only PMMA. As seen in Fig. S8, the PMMA coating film exhibited a similar *τ*_ave_ of 6.75 ns but a lower carrier extraction efficiency at the charge-selective interfaces compared to those of the gradient film. These results indicate that the improvement in *τ*_ave_ was mainly caused by the surface passivation effect of PMMA [56], and the improvement in the charge extraction efficiency can be attributed to the formation of a vertical Sn^2+^-gradient architecture. Additionally, we measured the dark current of the FASnI_3_ perovskite layer sandwiched between the two electrodes to characterize the difference in the electronic properties induced by the self-doping effect (Fig. 4f). The control sample showed a symmetric current plot at the positive and negative biases, whereas the gradient sample exhibited an asymmetric current plot with a much larger current output at the positive bias, indicating the formation of a depletion layer inside the gradient FASnI_3_ absorber, further confirming the formation of a built-in electric field induced by the vertical Sn^2+^ gradient.

### Oxidation Property of the FASnI_3_ Gradient Film

We investigated the effect of the vertical Sn^2+^ gradient on the oxidation properties of the FASnI_3_ absorber by tracking the changes in the Sn 3*d* XPS spectra before and after 3 h aging in air. As seen in Fig. S9a-b, approximately 76% of the Sn^2+^ cations were oxidized to Sn^4+^ in the FASnI_3_ control film, whereas 53% of the Sn^2+^ cations were oxidized to Sn^4+^ (Fig. S9c-d) in the gradient films. We deduced that this slower oxidation process could be due to the Sn-rich environment of the tin perovskite crystal significantly increasing the chemical potential of elemental Sn (*µ*_Sn_), which in turn increases the formation energy of Sn vacancies and therefore retards the oxidation reaction from Sn^2+^ to Sn^4+^ and Sn vacancies [39, 57].

### Performance of the Tin Perovskite Solar Cells

**Fig. 5:**
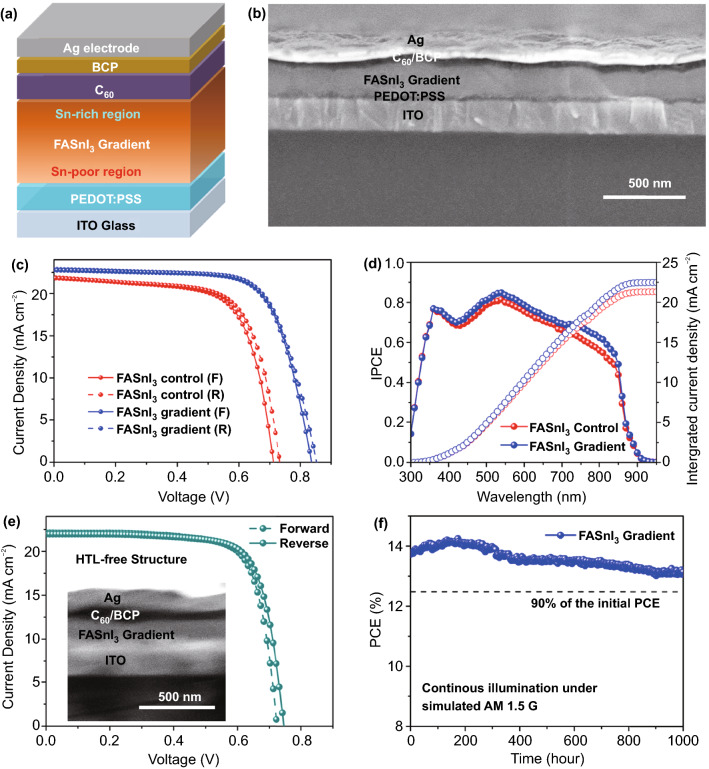
**a** Schematic illustration of the gradient FASnI_3_-based inverted PSCs and **b** the corresponding device cross-sectional SEM image. **c** Forward and reverse *J–V* curves of the best-performing FASnI_3_ control and gradient PSCs. **d** IPCE spectra and the integrated current density of the FASnI_3_ control and gradient devices. **e** Forward and reverse *J–V* curves of the HTL-free FASnI_3_ gradient device (the inset shows the cross-section of the SEM image). **f** Operational stability test of the encapsulated FASnI_3_ gradient PSC under simulated AM 1.5G light in air

To study the effect of the gradient FASnI_3_ absorber on the PV performance of tin PSCs, we fabricated inverted planar solar cells with an ITO/ PEDOT:PSS/FASnI_3_ gradient absorber/C_60_/BCP/Ag electrode structure (Fig. 5a). The cross-sectional SEM image of the FASnI_3_ gradient inverted PSCs (Fig. 5b) shows that a compact FASnI_3_ film with an average thickness of 230 nm was formed on the ITO/PEDOT:PSS substrate, while a 50-nm-thick electron-selective C_60_/BCP bilayer was deposited atop the FASnI_3_ perovskite. For comparison, the same structure was applied to the FASnI_3_ control cell with a 226-nm-thick perovskite absorber (the cross-sectional SEM image is shown in Fig. S10). We first compared the current density–voltage (*J–V*) curves of the FASnI_3_ control device, FASnI_3_ gradient device, device with only DMSO-vapor treatment, and device with only PMMA coating, as shown in Figs. 5c and S11a (the corresponding PV parameters are summarized in Tables 1 and S2). The PCEs of the control device were calculated as 10.52% and 10.89%, with short-circuit current density (*J*_SC_) of 21.88 and 21.81 mA cm^−2^, *V*_OC_ of 0.71 and 0.73 V, and fill factor (FF) of 67.7% and 68.4% under the forward and reverse scans, respectively. In contrast, the FASnI_3_ gradient devices showed much higher PCEs of 13.61% and 13.82%, with *J*_SC_ of 22.87 and 22.74 mA cm^−2^, *V*_OC_ of 0.84 and 0.85 V, and FF of 70.9% and 71.5% under the forward and reverse scans, respectively. These PCE values of the gradient FASnI_3_ PSCs were comparable with those of the state-of-the-art tin PSCs in previous studies (the PV parameters are summarized in Table S3). In contrast, the FASnI_3_ PSCs with only DMSO-vapor treatment showed a negligible efficiency improvement (10.87% under forward scan and 11.07% under reverse scan), and the Tof–SIMS two-dimensional mapping profile indicated a uniform vertical Sn^2+^ distribution in the perovskite absorber (Fig. S11b). These results revealed that DMSO-induced recrystallization without PMMA assistance could not create a Sn^2+^ gradient to enhance the electric field and boost the tin PSC performance. In addition, the PSCs with only PMMA coating showed PCEs of 11.36% (forward scan) and 11.65% (reverse scan) with a slight increase in *V*_OC_ (approximately 40 mV). The above analysis indicates that the *J*_SC_ improvement in the FASnI_3_ gradient device was induced by the Sn^2+^-gradient architecture with an enhanced electric field, and the *V*_OC_ improvement was caused by the synergistic effect of the PMMA passivation and Sn^2+^-gradient architecture.

**Table 1:** The PV parameters of the FASnI_3_ control, FASnI_3_ gradient, FASnI_3_ with the surface passivation of PMMA, as well as the HTL-free FASnI_3_ gradient PSCs

Samples	Scan Direction	*J*_SC_ (mA cm^−2^)	*V*_OC_ (V)	FF (%)	PCE (%)
FASnI_3_ control	Forward	21.88	0.71	67.7	10.52
	Reverse	21.81	0.73	68.4	10.89
FASnI_3_ gradient	Forward	22.87	0.84	70.9	13.61
	Reverse	22.74	0.85	71.5	13.82
FASnI_3_ gradient (HTL-free structure)	Forward	21.21	0.73	71.9	11.61
	Reverse	21.39	0.75	72.3	11.91

To confirm the reliability of the *J–V* measurements, the IPCE spectra of the control and gradient FASnI_3_ PSCs were measured (Fig. 5d). The integrated *J*_SC_ of the FASnI_3_ control and gradient cells were calculated to be 21.39 and 22.47 mA cm^−2^, respectively. These *J*_SC_ values were close to those of the *J–V* measurements. In addition, we found that the IPCE increased significantly in the range of 700–850 nm when a gradient FASnI_3_ absorber was incorporated, this IPCE increase of gradient FASnI_3_ was associated with improvement in carrier extraction efficiency at the back interface, which is caused by the large downward band bending at the gradient FASnI_3_/C_60_ interface that promotes the electron extraction process.

To study the reproducibility of the FASnI_3_ control and gradient PSCs, we fabricated 40 cells for each kind of device. The control devices showed an average PCE of 9.83% with a standard deviation of 0.62% (Fig. S12). In contrast, the average PCE of the FASnI_3_ gradient device was calculated to be 13.12%, with a smaller standard deviation of 0.49%. These results further confirmed the high reproducibility of the PMMA-assisted recrystallization method for fabricating high-performance tin PSCs.

In addition, Mott–Schottky analysis was applied to investigate the built-in potential in the FASnI_3_ control and gradient PSCs. As shown in Fig. S13, we derived the built-in potential results from the Mott–Schottky plots by calculating their intercepts at the x-axis and found that the built-in potential of the gradient tin PSCs was approximately 0.14 V higher than that of the control PSCs, which was consistent with the *V*_OC_ improvement demonstrated by the *J–V* tests (Fig. 5c).

We further fabricated an HTL-free FASnI_3_ gradient PSC to emphasize its unique charge separation capability by directly removing the PEDOT:PSS layer. As seen in Fig. 5e, the HTL-free gradient device could still maintain a similar photocurrent to the completed cell (the integrated *J*_SC_ was calculated as 22.13 mA cm^−2^ in the IPCE spectrum shown in Fig. S14) and exhibited efficiencies of 11.61% and 11.91% with FF values of 71.9% and 72.3% under the forward and reverse scans, respectively. The higher FF was attributed to the reduced series resistance for hole transport in the HTL-free PSCs. It is worth noting that this is the highest PCE value reported for HTL-free tin PSCs to date. (A comparison of the HTL-free device performance between this and previous studies is presented in Table S4.)

Stability is another important criterion for the commercialization of tin PSCs. In the present study, we tracked the operational stability of the best-performing FASnI_3_ gradient cell under simulated AM 1.5G light (100 mW cm^−2^) at the maximum power point in air. As shown in Fig. 5f, the FASnI_3_ gradient cell exhibited an initial power output of 13.80%. Interestingly, the power output gradually increased to over 14% at an operational time of 100 h and this high power output was maintained for approximately 200 h before an obvious PCE drop. This PCE improvement induced by the light-soaking effect will be further investigated in the future. After 1,000 h of operation, we found that the PCE of the FASnI_3_ gradient cell was maintained at over 13%, indicating that the surface Sn-rich region could serve as a protective layer to suppress oxygen-induced degradation of tin perovskite crystals. For comparison, we also measured the operational stability of the FASnI_3_ control PSCs and observed a large PCE loss of more than 20% after 500 h operation at the maximum power point (Fig. S15), which was associated with the relatively poor long-term stability of the control device. In addition, we measured the thermal stabilities of the control and gradient cells. As shown in Fig. S16, both devices showed an efficiency loss of approximately 5% after heating at 60 °C in a N_2_ atmosphere for 100 h, indicating that such an inverted device structure is stable under thermal aging.

## Conclusion

In summary, we propose a novel recrystallization strategy to fabricate a gradient FASnI_3_ perovskite absorber with an enhanced built-in electric field to minimize both bulk and surface charge recombination losses in tin PSCs. The top Sn-rich and bottom Sn-poor regions caused a large *E*_F_ difference, which generated an additional electric field inside the tin perovskite layer to separate the photo-induced electrons and holes. As a result, the FASnI_3_ absorber with a vertical Sn^2+^ gradient exhibited a promising efficiency of 13.82% for inverted FASnI_3_-based PSCs, which is one of the highest PCEs reported for lead-free perovskite solar cells to date. Gradient tin PSCs are ultrastable, maintaining over 13% efficiency after operation at 1-sun illumination for 1,000 h. More importantly, the present study provides a universal way to further improve the performance of tin PSCs in the future because it is suitable for all kinds of Sn or mixed Sn–Pb perovskite compositions.

## Supplementary Information

Below is the link to the electronic supplementary material.

Supplementary file1 (PDF 1628 KB)
