# Supplementary material for: Heterogeneous FASnI3 Absorber with Enhanced Electric Field for High-Performance Lead-Free Perovskite Solar Cells
Source: Nanomicro Lett. 2022 Apr 8;14:99. doi: 10.1007/s40820-022-00842-4 (PMC8993987; doi:10.1007/s40820-022-00842-4)
Supplement: Supplementary file 1 — Supplementary file1 (PDF 1628 KB) [file 40820_2022_842_MOESM1_ESM.pdf]

Supporting Information for

Heterogeneous $FASnI_3$ Absorber with Enhanced Electric Field for High-Performance Lead-Free Perovskite Solar Cells

Tianhao Wu[1, 3], Xiao Liu[2, *], Xinhui Luo[1], Hiroshi Segawa[2], Guoqing Tong[3], Yiqiang Zhang[4], Luis K. Ono[3], Yabing Qi[3], and Liyuan Han[1, 2, *]

[1]State Key Laboratory of Metal Matrix Composites, School of Material Science and Engineering, Shanghai Jiao Tong University, Shanghai 200240, P. R. China

[2]Special Division of Environmental and Energy Science, Komaba Organization for Educational Excellence (KOMEX), College of Arts and Sciences, University of Tokyo, Tokyo 153-8902, Japan

[3]Energy Materials and Surface Sciences Unit (EMSSU), Okinawa Institute of Science and Technology Graduate University (OIST), 1919-1 Tancha, Onna-son, Kunigami-gun, Okinawa 904-0495, Japan

[4]School of Materials Science and Engineering, Henan Institute of Advanced Technology, Zhengzhou University, Zhengzhou 450001, P. R. China

*Corresponding authors. E-mail: liu.xiao@mail.u-tokyo.ac.jp (Xiao Liu); han.liyuan@sjtu.edu.cn (Liyuan Han)

S1 Chemicals

All chemicals and reagents were used as received from commercial sources without further purification, including PEDOT:PSS (Clevious P VP Al 4083), SnI_2 (99.99%, Sigma-Aldrich), Formamidinium iodide (FAI) (98%, Tokyo Chemical Industry Co., Japan), ,SnF_2 (99%, Sigma-Aldrich), C_{60} (99.5%, Sigma-Aldrich), Bathocuproine (99%, FUJIFILM Wako), Poly(methyl methacrylate) (PMMA, Sigma). All solutions were filtered through a 0.2-μm filter before use.

S2 Supplementary Figures and Tables

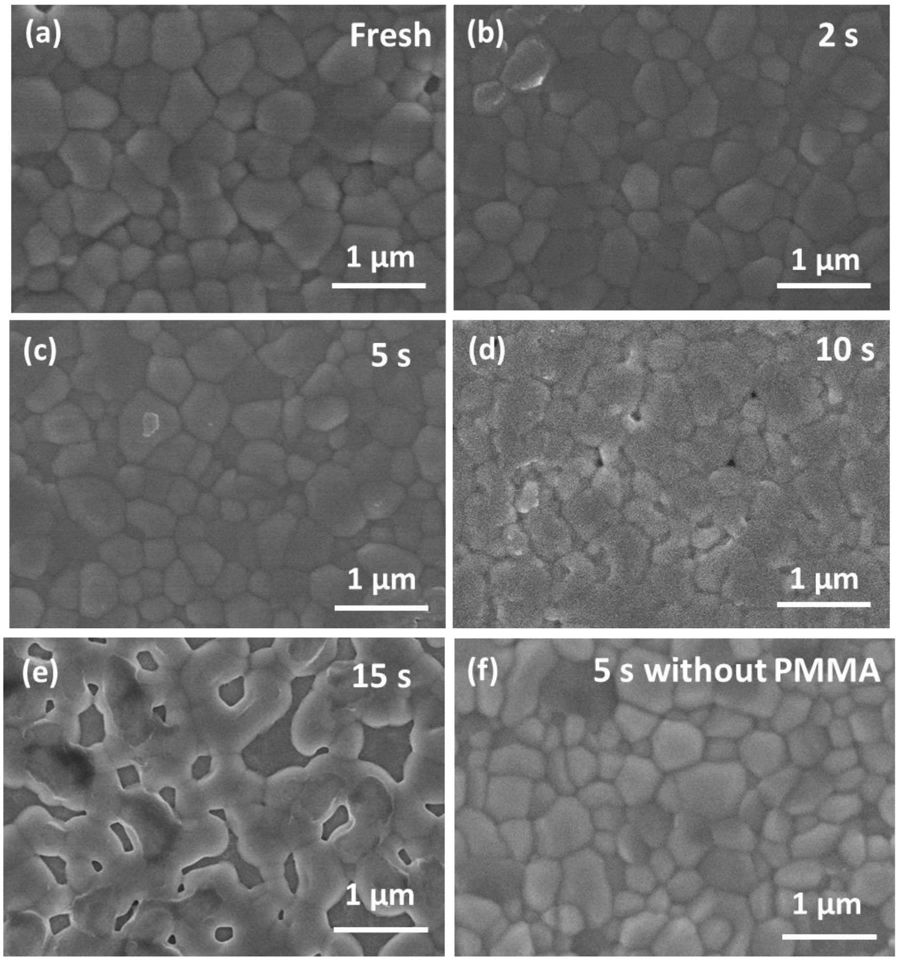

Fig. S1 Top-view scanning electron microscope (SEM) images of the polymethyl methacrylate (PMMA)-coated $FASnI_3$ perovskite films **a** before and after exposed to the dimethyl sulfoxide (DMSO) vapor for **b** 2 s, **c** 5 s, **d** 10 s, and **e** 15 s, following by thermal annealing at 100 °C for recrystallization process. **f** Top-view SEM image of the $FASnI_3$ film without PMMA coating after exposed to the DMSO vapor for 5 s, following by annealing at 100 °C for recrystallization

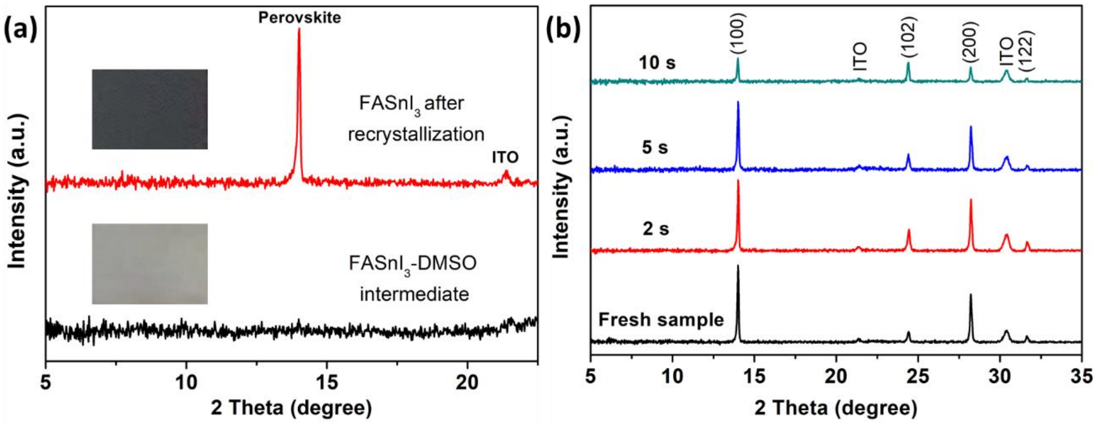

Fig. S2 a X-ray diffraction (XRD) patterns of the $FASnI_3$-DMSO intermediate film with PMMA coating after 5 s DMSO treatment and the recrystallized $FASnI_3$ film with PMMA coating. The insets show the photographs of the films. **b** The XRD patterns of the PMMA-coated $FASnI_3$ fresh film and the films exposed to the DMSO vapor for 2 s, 5 s, and 10 s following by thermal annealing at 100 °C to remove the DMSO solvent

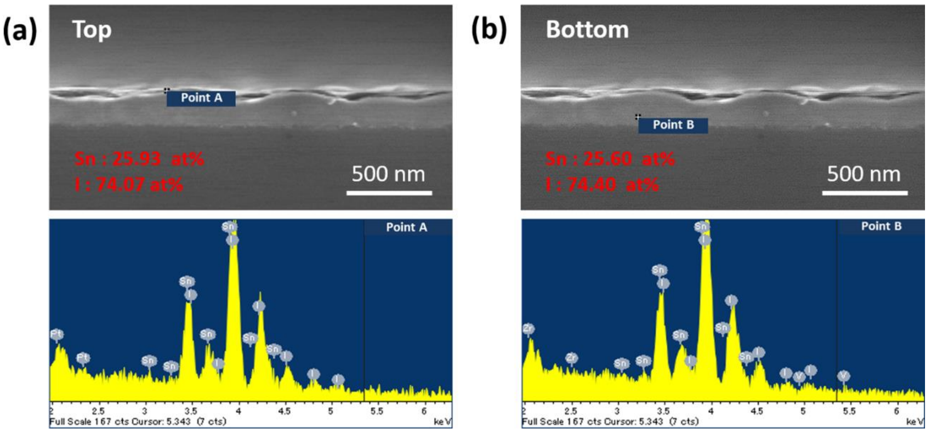

Fig. S3 The cross-section SEM images and the corresponding EDS point-scanning profiles of the **a** top and **b** bottom of $FASnI_3$ control film

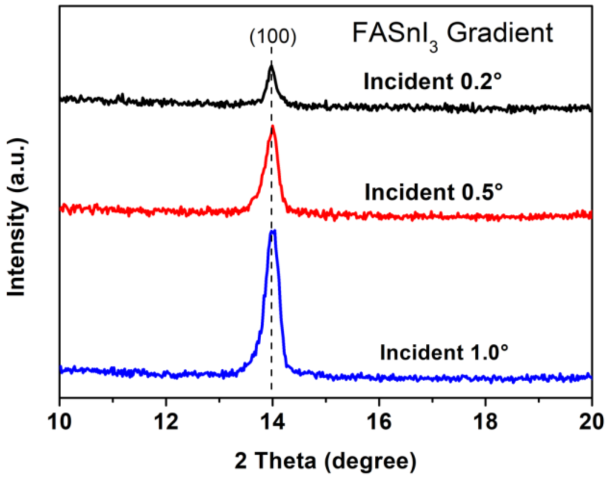

Fig. S4 The grazing incident X-ray diffraction (GIXRD) patterns of the $FASnI_3$ gradient film measured with the incident angles changing from 0.2° to 1.0°

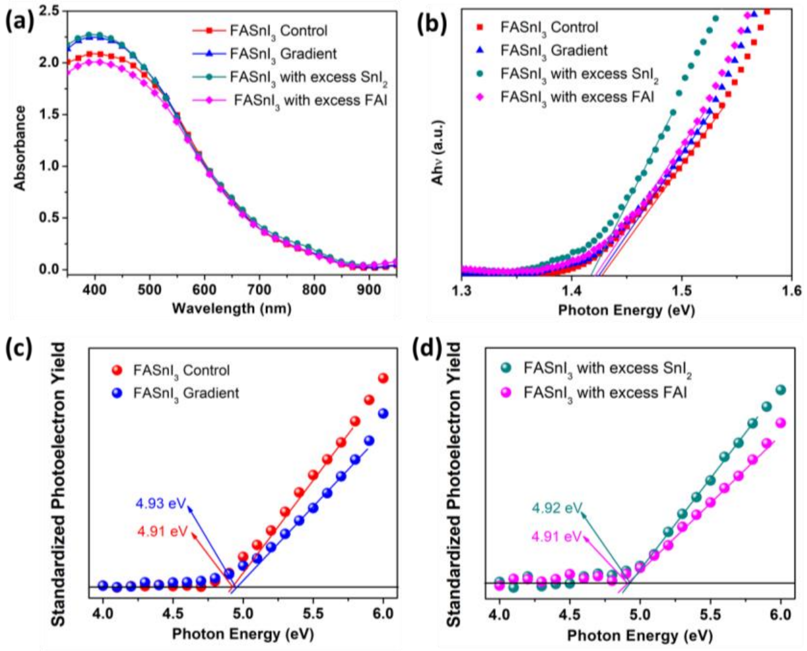

Fig. S5 a UV-vis absorption spectrums and **b** Tauc plots of the $FASnI_3$ control film, $FASnI_3$ gradient film, and the $FASnI_3$ films with excess SnI_2 or FAI. **c** The PESA plots of the $FASnI_3$ control and gradient perovskite films. **d** The PESA plots of the $FASnI_3$ films with excess SnI_2 (7.7 mol%) or FAI (6.3 mol%)

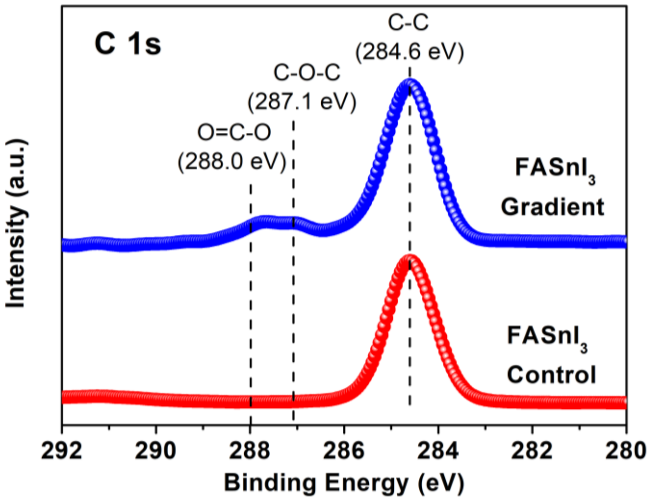

Fig. S6 XPS high-resolution C 1s spectrums of the $FASnI_3$ control and gradient perovskite films. The peaks at 284.6 eV, 287.1 eV, and 288.0 eV are associated with the C-C, C-O-C, and O=C-O groups, respectively [S1]. The C-O-C and O=C-O signals indicate the presence of PMMA in $FASnI_3$ gradient film

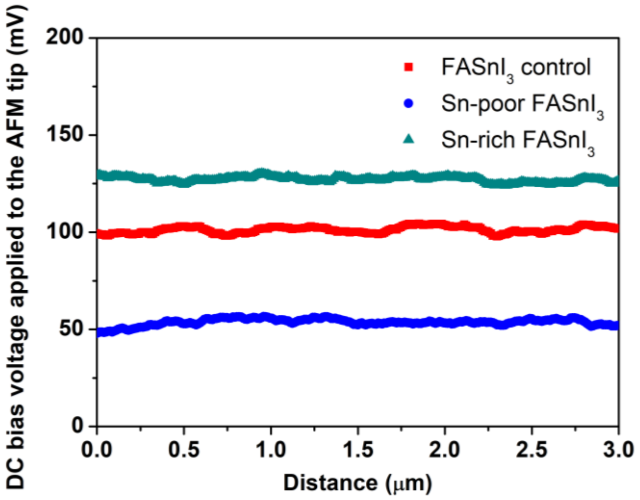

Fig. S7 Surface potential line profiles of the three types of corresponding tin perovskite samples. Note that the recorded value is the DC bias voltage applied to the AFM tip to nullify the contact potential difference between the AFM tip and the sample surface

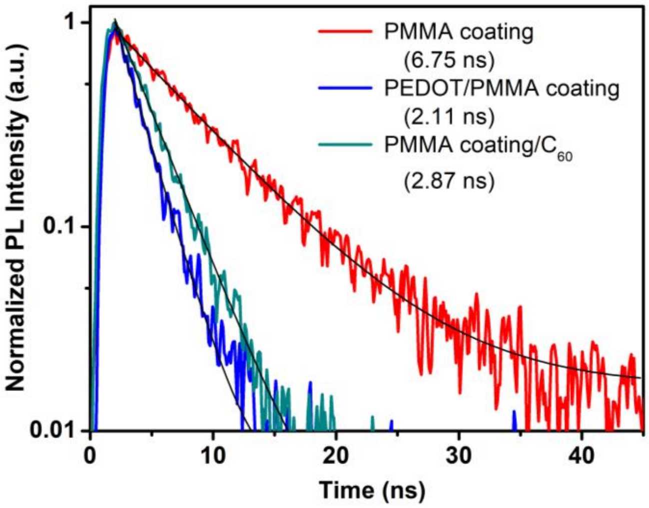

Fig. S8 TRPL spectra of the $FASnI_3$ film with PMMA coating deposited on quartz substrate before and after contacted with the C_{60} or PEDOT: PSS layers

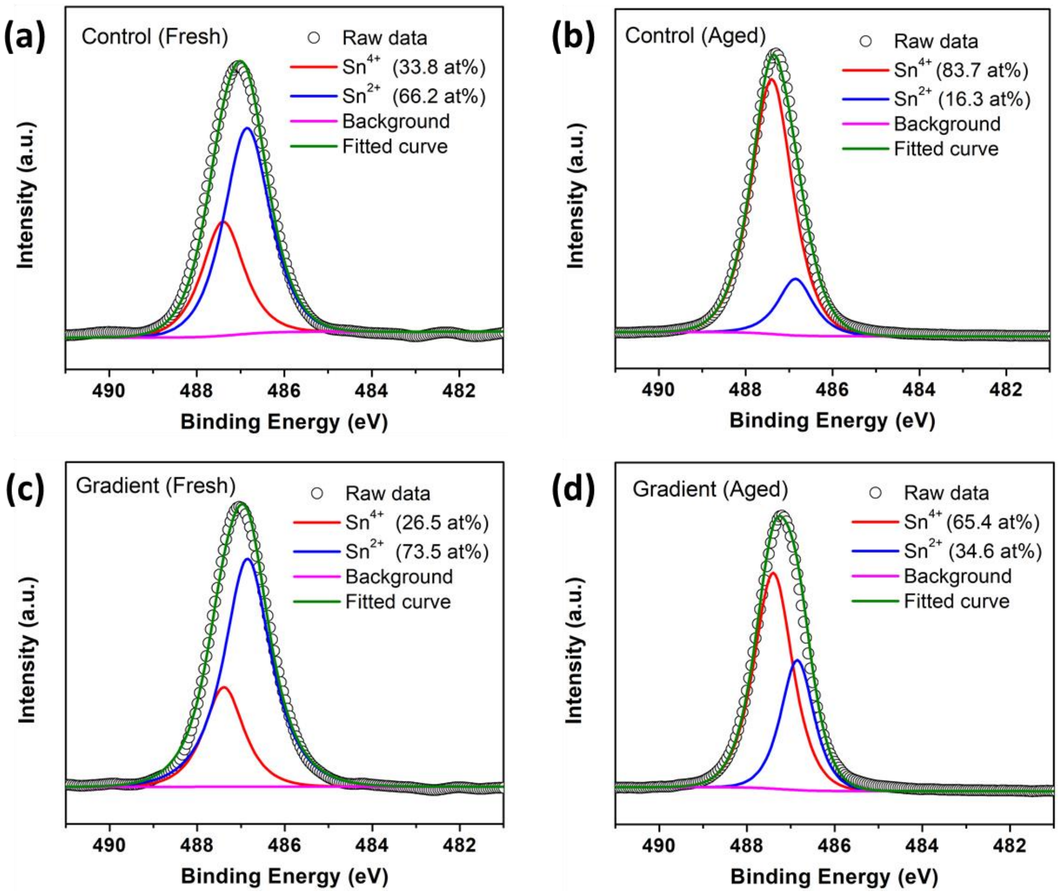

Fig. S9 The XPS high-resolution Sn 3d spectrums of the $FASnI_3$ control film **a** before and **b** after being exposed to air for 3 h, and the $FASnI_3$ gradient film **c** before and **d** after the aging process. The fitting peaks at 486.85 and 487.50 eV are associated with the Sn^{2+} and Sn^{4+} components, respectively [S2]

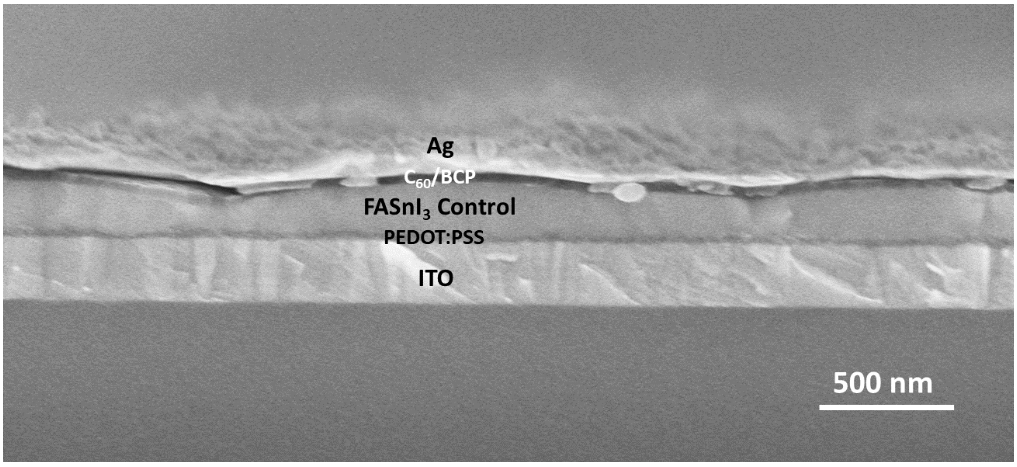

Fig. S10 The cross-section SEM image of the control device with a structure of ITO/PEDOT:PSS/$FASnI_3$ control (226 nm)/C_{60}/ bathocuproine (BCP)/Ag electrode

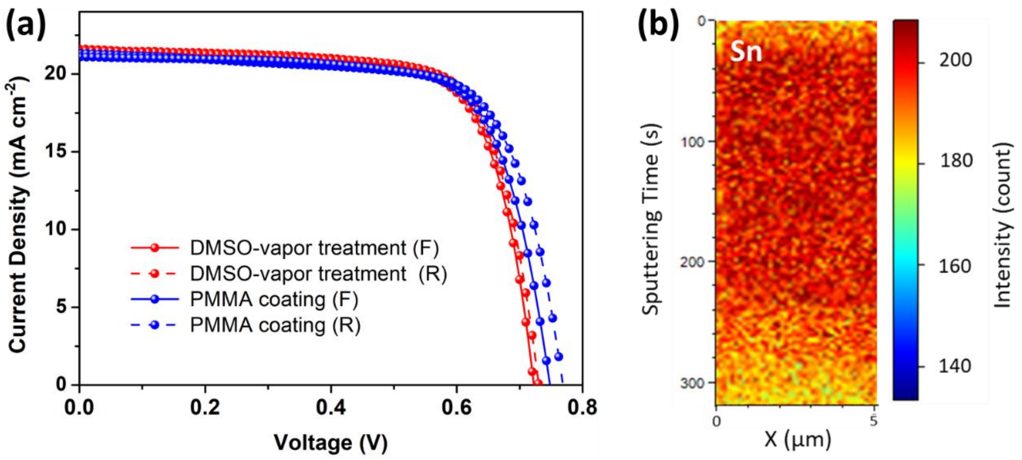

Fig. S11 a *J-V* curves of the best-performing $FASnI_3$ PSCs with DMSO-vapor treatment and with PMMA coating. **b** Tof-SIMS 2D mapping profiles of Sn along the *x-z* plane for the $FASnI_3$ film with DMSO-vapor treatment

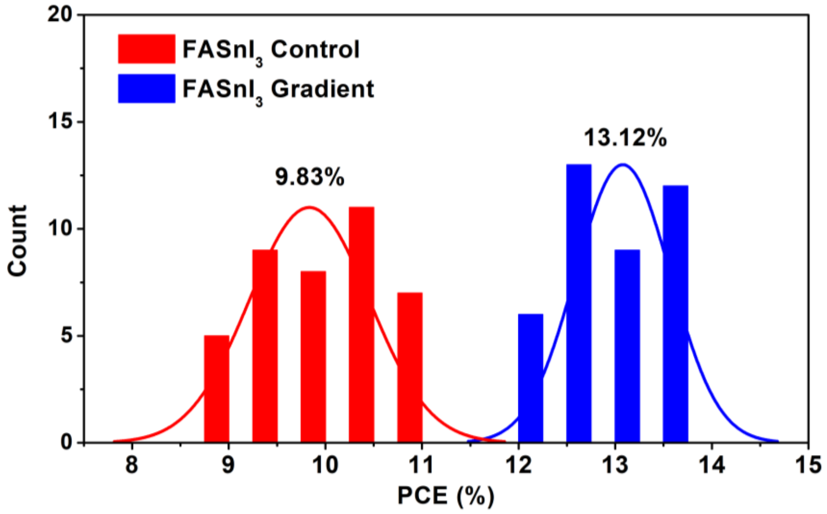

Fig. S12 Histogram of the PCE distribution based on 40 individual cells for the $FASnI_3$ control and $FASnI_3$ gradient PSCs

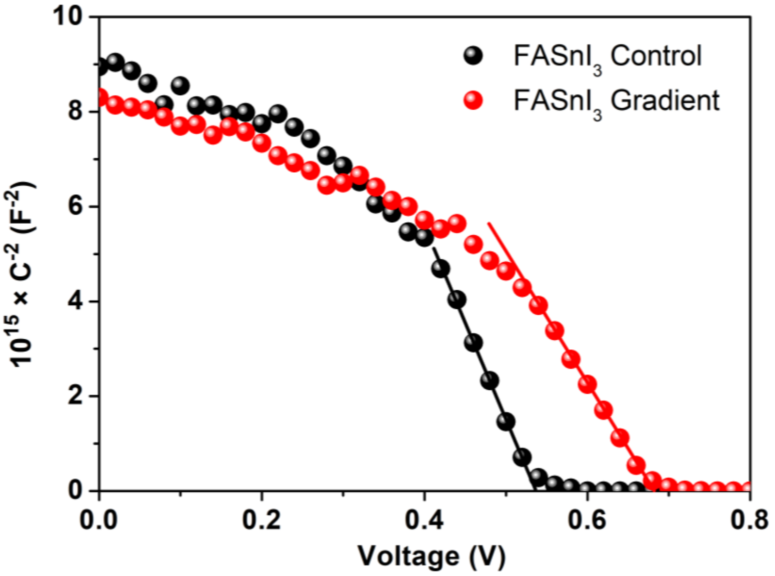

Fig. S13 Mott-Schottky plots of the $FASnI_3$ control and $FASnI_3$ gradient PSCs

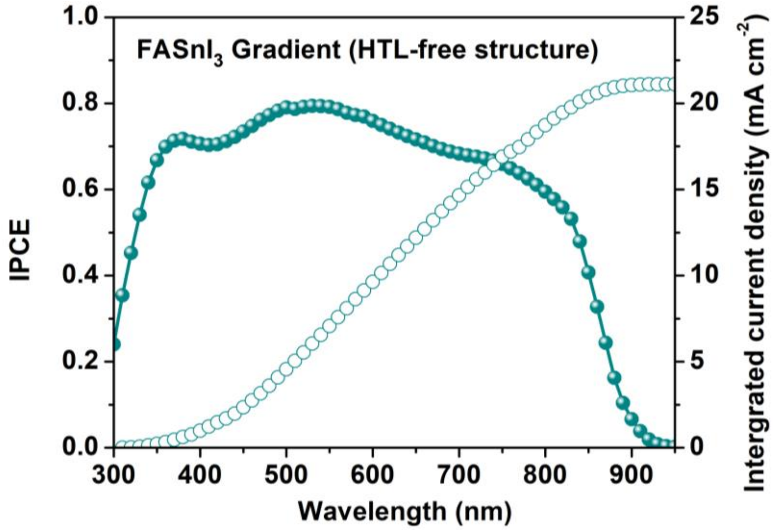

Fig. S14 Incident photon-to-current efficiency (IPCE) spectrum of the $FASnI_3$ gradient PSC with a HTL-free structure

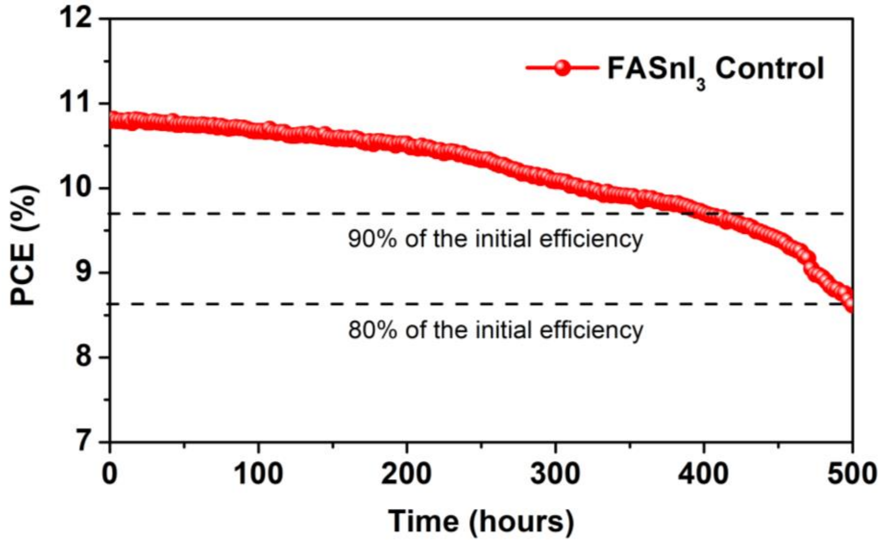

Fig. S15 Long-term operational stability test of the $FASnI_3$ control PSCs with encapsulation under continuous light soaking (AM 1.5 G, 100 mW cm^{-2}) in air (about 20% humidity)

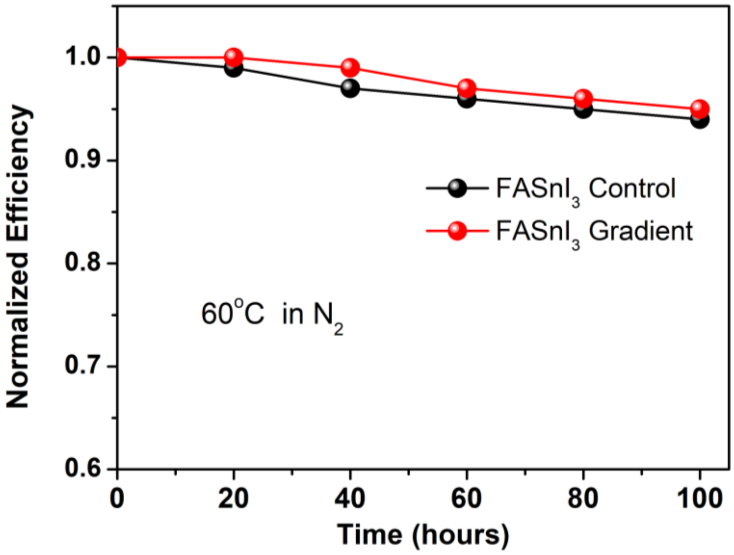

Fig. S16 Thermal stability test of the $FASnI_3$ control and gradient PSCs without encapsulation under 60 °C heating in a N_2 glove box

Table S1 The decay lifetime of time-resolved photoluminescence (PL) curves for the corresponding tin perovskite samples derived from the double-exponential decay equation: $y=A_1\exp(-t/\tau_1) + A_2\exp(-t/\tau_2)$. Where y is the PL intensity, t is the decay time, A_1 and A_2 are the two independent constants. The decay lifetime τ_1 and τ_2 could be determined by the decay time relative to PL intensity dropping to 1/e of its initial value. The proportion of these two decay lifetimes is calculated by $A_1/(A_1+A_2)$ for τ_1 and $A_2/(A_1+A_2)$ for τ_2

Sample	τ_1			τ_2			τ_{ave}
	Value (ns)	Error (ns)	Ratio (%)	Value (ns)	Error (ns)	Ratio (%)	(ns)
$FASnI_3$ control	0.59	0.05	0.8	4.85	0.13	99.2	4.82
PEDOT/$FASnI_3$ control	0.36	0.02	46.5	3.15	0.10	53.5	1.85
$FASnI_3$ control/C_{60}	0.47	0.02	33.8	3.48	0.11	66.2	2.46
$FASnI_3$ gradient	0.43	0.06	0.9	7.04	0.15	99.1	6.98
PEDOT/$FASnI_3$ gradient	0.22	0.02	37.3	1.65	0.07	62.7	1.05
$FASnI_3$ gradient/C_{60}	0.32	0.03	47.9	2.07	0.12	52.1	1.23
PMMA coating	0.55	0.06	0.8	6.80	0.18	99.2	6.75
PEDOT/PMMA coating	0.72	0.09	52.2	3.63	0.13	47.8	2.11
PMMA coating/C_{60}	0.89	0.08	42.5	4.33	0.15	57.5	2.87

Table S2 The PV parameters of the $FASnI_3$ control, $FASnI_3$ gradient, and the HTL-free $FASnI_3$ gradient tin PSCs

Samples	Scan Direction	J_{SC} (mA cm^{-2})	V_{OC} (V)	FF (%)	PCE (%)
Recrystallization only	forward	21.35	0.71	71.7	10.87
	reverse	21.24	0.72	72.4	11.07
PMMA only	forward	21.31	0.75	71.1	11.36
	reverse	21.14	0.77	71.6	11.65

Table S3 Summary of the reported photovoltaic parameters of the state-of-the-art tin PSCs with over 13% efficiency

Perovskite Component	J_{SC} (mA cm^{-2})	V_{OC} (V)	FF (%)	PCE (%)	Refs.
$FASnI_{2.85}Br_{0.1}Cl_{0.05}$	23.02	0.81	72.0	13.40	[S3]
$FA_{0.9}EA_{0.1}SnI_3$[a]	20.32	0.84	78.0	13.24	[S4]
$FA_{0.8}GA_{0.2}SnI_3$[b]	21.90	0.81	76.0	13.50	[S5]
$PEA_{0.15}FA_{0.85}SnI_{2.55}Br_{0.45}$[c]	20.60	0.91	77.1	14.63	[S6]
$FA_{0.75}MA_{0.25}SnI_3$	24.90	0.77	76.7	14.70	[S7]
$FPEA_{0.1}FA_{0.9}SnI_{2.9}Br_{0.1}$[d]	24.91	0.84	71.7	14.81	[S8]
$FASnI_3$	22.74	0.85	71.5	13.82	This work

[a]EA indicates the ethylammonium cation; [b]GA indicates the guanidinium cation; [c]PEA indicates the phenethylammonium cation; [d]FPEA indicates the 4-fluoro-phenethylammonium cation

Table S4 Summary of the reported photovoltaic parameters of the HTL-free tin PSCs

Perovskite Component	J_{SC} (mA cm^{-2})	V_{OC} (V)	FF (%)	PCE (%)	Refs.
$CsSnI_3$	9.89	0.50	68.0	3.35	[S9]
$TEA_xFA_{1-x}SnI_3$[a]	17.05	0.50	61.2	5.17	[S10]
$FASnI_3$	22.03	0.67	68.5	10.11	[S11]
$FASnI_3$	21.39	0.75	72.3	11.91	This work

[a]TEA indicates the thienylethylammonium cation

Supplementary References

[S1] X. Liu, Y. Cheng, C. Liu, T. Zhang, N. Zhang et al., 20.7% highly reproducible inverted planar perovskite solar cells with enhanced fill factor and eliminated hysteresis. Energy Environ. Sci. **12**(5), 1622-1633 (2019). https://doi.org/10.1039/C9EE00872A

[S2] F. Wang, J. Ma, F. Xie, L. Li, J. Chen et al., Organic cation-dependent degradation mechanism of organotin halide perovskites. Adv. Funct. Mater. **26**(20), 3417-3423 (2016). https://doi.org/doi:10.1002/adfm.201505127

[S3] C. Wang, Y. Zhang, F. Gu, Z. Zhao, H. Li et al., Illumination durability and high-efficiency Sn-based perovskite solar cell under coordinated control of phenylhydrazine and halogen ions. Matter **4**(2), 709-721 (2021). https://doi.org/10.1016/j.matt.2020.11.012

[S4] K. Nishimura, M.A. Kamarudin, D. Hirotani, K. Hamada, Q. Shen et al., Lead-free tin-halide perovskite solar cells with 13% efficiency. Nano Energy **74**, 104858 (2020). https://doi.org/10.1016/j.nanoen.2020.104858

[S5] E. Jokar, H.S. Chuang, C.H. Kuan, H.P. Wu, C.H. Hou et al., Slow passivation and inverted hysteresis for hybrid tin perovskite solar cells attaining 13.5% via sequential deposition. J. Phys. Chem. Lett. **12**(41), 10106-10111 (2021). https://doi.org/10.1021/acs.jpclett.1c03107

[S6] X. Jiang, H. Li, Q. Zhou, Q. Wei, M. Wei et al., One-step synthesis of $SnI_2\cdot(DMSO)_x$ adducts for high-performance tin perovskite solar cells. J. Am. Chem. Soc. **143**(29), 10970–10976 (2021). https://doi.org/10.1021/jacs.1c03032

[S7] J. Zhou, M. Hao, Y. Zhang, X. Ma, J. Dong et al., Chemo-thermal surface dedoping for high-performance tin perovskite solar cells. Matter **5**(2), 683-693 (2022). https://doi.org/10.1016/j.matt.2021.12.013

[S8] B.B. Yu, Z. Chen, Y. Zhu, Y. Wang, B. Han et al., Heterogeneous 2D/3D tin-halides perovskite solar cells with certified conversion efficiency breaking 14%. Adv. Mater. **33**(36), 2102055 (2021). https://doi.org/10.1002/adma.202102055

[S9] K.P. Marshall, M. Walker, R.I. Walton, R.A. Hatton, Enhanced stability and efficiency in hole-transport-layer-free $CsSnI_3$ perovskite photovoltaics. Nat. Energy **1**, 16178 (2016). https://doi.org/10.1038/nenergy.2016.178

[S10] J.T. Lin, T.C. Chu, D.G. Chen, Z.X. Huang, H.C. Chen et al., Vertical 2D/3D heterojunction of tin perovskites for highly efficient HTL-free perovskite solar cell. ACS Appl. Energy Mater. **4**(3), 2041-2048 (2021). https://doi.org/10.1021/acsaem.0c02451

[S11] X. Liu, T. Wu, C. Zhang, Y. Zhang, H. Segawa et al., Interface energy-level management toward efficient tin perovskite solar cells with hole-transport-layer-free structure. Adv. Funct. Mater. **31**(50), 2106560 (2021). https://doi.org/10.1002/adfm.202106560
